# The clinical chameleon: eosinophilic myocarditis presenting as acute coronary syndrome—a case report

**DOI:** 10.1093/ehjcr/ytag752

**Published:** 2026-10-12

**Authors:** Denise Belén Magnifico, Ignacio Melem, Sergio Baratta, Renzo Melchiori

**Affiliations:** Department of Cardiology, Hospital Universitario Austral, Avenida Juan Domingo Perón 1500, B1629AHJ Pilar, Buenos Aires, Argentina; Department of Cardiology, Hospital Universitario Austral, Avenida Juan Domingo Perón 1500, B1629AHJ Pilar, Buenos Aires, Argentina; Department of Cardiology, Hospital Universitario Austral, Avenida Juan Domingo Perón 1500, B1629AHJ Pilar, Buenos Aires, Argentina; Department of Cardiology, Hospital Universitario Austral, Avenida Juan Domingo Perón 1500, B1629AHJ Pilar, Buenos Aires, Argentina

**Keywords:** Eosinophilic myocarditis, MINOCA (Myocardial infarction with non-obstructive coronary arteries), Cardiac magnetic resonance, Intracavitary thrombus, Multimodality imaging, Case report

## Abstract

**Background:**

Eosinophilic myocarditis is a rare inflammatory cardiomyopathy that may clinically mimic acute coronary syndrome and should be considered in patients with non-obstructive coronary arteries, often resulting in diagnostic delay.

**Case summary:**

A 27-year-old man presented with recurrent chest pain and was initially misdiagnosed with a panic attack. On admission, laboratory evaluation revealed marked peripheral eosinophilia (1800/mm^3^), mildly elevated troponin, and increased serum IgE levels. Echocardiography demonstrated reduced left ventricular ejection fraction and a large apical intracavitary thrombus. Coronary angiography revealed normal coronary arteries. Cardiac magnetic resonance imaging showed myocardial oedema and circumferential subendocardial late gadolinium enhancement consistent with eosinophilic myocarditis. Extensive aetiological workup was negative. Although endomyocardial biopsy showed non-specific findings, the diagnosis was established through multimodal integration of clinical, imaging, and laboratory data. High-dose corticosteroid therapy and anticoagulation resulted in rapid clinical improvement, recovery of ventricular function, and near-complete thrombus resolution.

**Discussion:**

Eosinophilic myocarditis should be considered in young patients presenting with chest pain and non-obstructive coronary arteries. Multimodality imaging—particularly cardiac magnetic resonance—plays a key role in diagnosis, while early corticosteroid therapy may lead to rapid clinical and functional recovery.

**Conclusion:**

This case underscores the importance of maintaining a high index of clinical suspicion for EM in young patients presenting with chest pain and peripheral eosinophilia. A multimodal diagnostic approach integrating cMRI findings, laboratory data, and exclusion of alternative aetiologies is essential when biopsy results are inconclusive. Early immunosuppressive therapy is key to reversing myocardial damage and preventing progression to end-stage heart failure.

Learning points

- To recognize eosinophilic myocarditis as a cause of chest pain with non-obstructive coronary arteries.
- To highlight the diagnostic value of multimodality imaging, particularly cardiac magnetic resonance.
- To emphasize the importance of early corticosteroid therapy to prevent irreversible myocardial damage.

## Introduction

Eosinophilic myocarditis (EM) is a rare but potentially life-threatening form of inflammatory cardiomyopathy characterized by eosinophilic infiltration of the myocardium. The condition may arise from various aetiologies, including drug hypersensitivity, parasitic infections, autoimmune diseases, and myeloproliferative disorders. Clinically, EM may mimic acute myocardial infarction, frequently leading to diagnostic delay. Early recognition is crucial, as prompt initiation of immunosuppressive therapy can prevent irreversible myocardial injury and improve clinical outcomes.

## Summary figure

**Figure:**
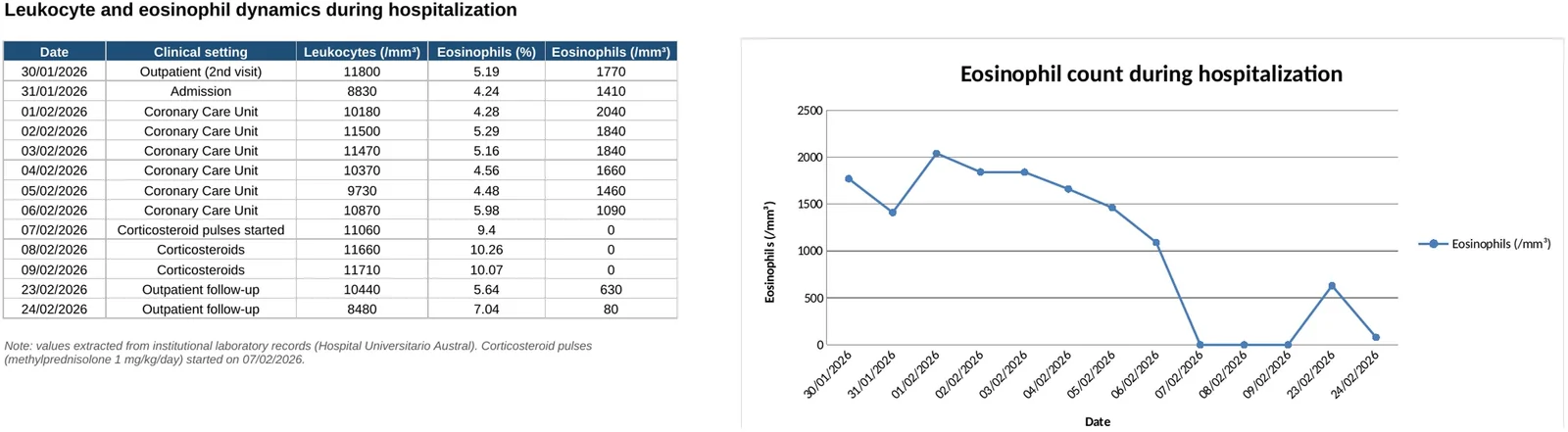


## Case presentation

On 2 February 2026, a 27-year-old overweight male patient was admitted, referred from another clinic, with a diagnosis of evolved acute myocardial infarction with the presence of an intracavitary thrombus in the left ventricle.

He reported that 4 months prior to his hospital admission, in October 2025, he experienced an episode of oppressive retrosternal pain radiating to the jaw, associated with dyspnoea in functional Class IV. On that occasion, he consulted a healthcare centre where an ECG and echocardiogram were performed, both of which showed no changes; the symptomatology was initially interpreted as a panic attack and he was treated with benzodiazepines.

However, the clinical picture progressed, prompting a new consultation on 30 January 2026. On this occasion, he presented with a new episode of oppressive retrosternal pain, of shorter duration and lesser intensity. A new echocardiogram was performed, which revealed a non-dilated left ventricle (LV) with an ejection fraction (EF) of 55%, apical akinesia, and a hyperechogenic image of 20 × 15 mm at the apex, compatible with an intracavitary thrombus, which was the reason for referral to our higher-complexity centre.

Upon admission, an electrocardiogram (ECG) was performed, which showed Q waves in V1 and V2, a non-specific intraventricular conduction delay, and negative T waves in DI, aVL, and V2–V6 (*Figure 1*).

**Figure 1:**
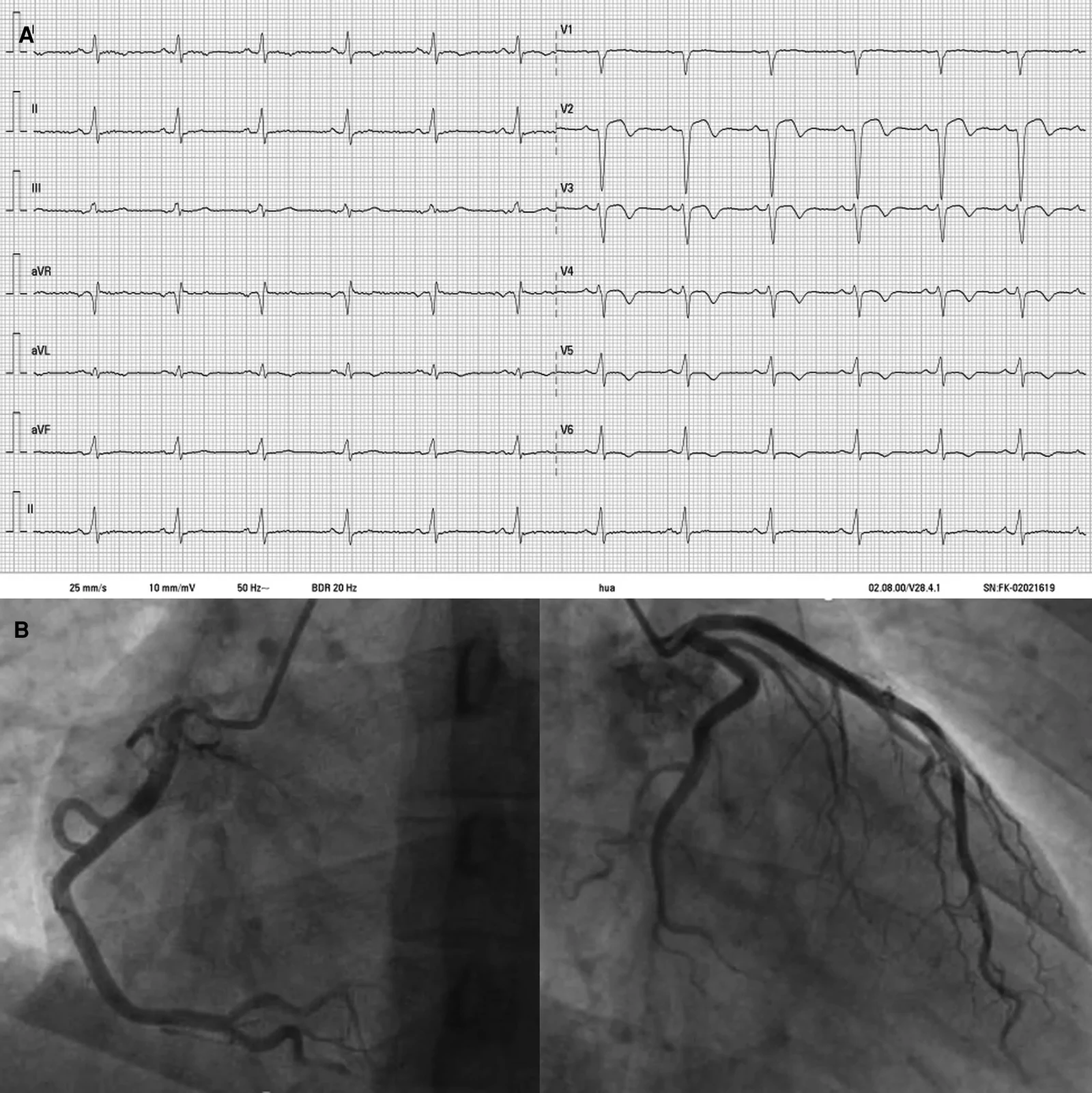
Initial diagnostic evaluation. *(A)* Admission electrocardiogram (ECG) showing sinus rhythm, heart rate of 75 b.p.m., preserved PR interval with a duration of 160 ms, widened QRS with a duration of 120 ms, and a non-specific intraventricular conduction delay, with lateral repolarization abnormality evidenced by inverted T waves in DI, aVL, and V2 through V6. *(B)* Coronary angiography. Left image: Cranial projection. Right coronary artery and posterior descending artery are visualized without significant angiographic lesions. Right image: Right anterior oblique projection with cranial angulation. Left anterior descending artery and circumflex artery are visualized without significant angiographic lesions.

On physical examination, the patient was haemodynamically stable, normotensive, without heart failure, and without new pain episodes. Initial laboratory studies revealed leucocytosis of 11 000/mm^3^ with marked peripheral eosinophilia of 15%, representing an absolute value of 1800/mm^3^ (Supplementary material online, Figure S1). Myocardial damage markers showed a troponin level of 19 pg/mL (reference value <34 pg/mL) and a proBNP of 325 pg/mL (reference value <125 pg/mL). A new echocardiogram was performed, which demonstrated an EF of 35%–40% with apical inferior, apical, apical lateral, and apical septal akinesia, and severe medial anteroseptal hypokinesia. Additionally, a mobile, hyperechogenic image of 30 × 20 mm was observed at the apical level, adherent to akinetic segments, compatible with an intracavitary thrombus (*Figure 2*; Supplementary material online, Videos S1 and S2).

**Figure 2:**
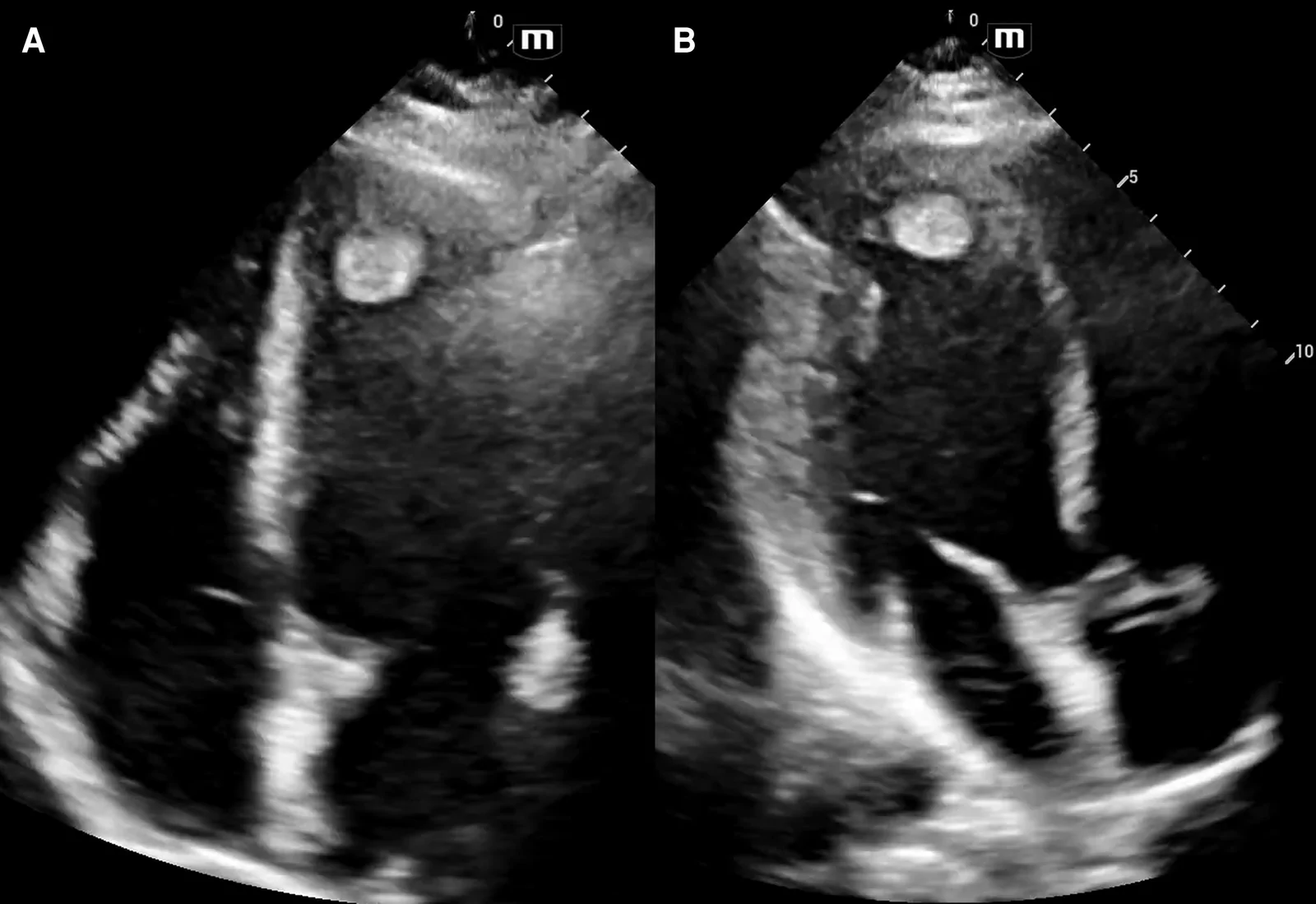
Admission echocardiogram. *(A)* Apical four-chamber echocardiographic view; *(B)* apical three-chamber echocardiographic view. Both images demonstrate cardiac chambers of normal dimensions, with a hyperechogenic image adherent to the apical region, in relation to the akinetic segments.

In this context, treatment was initiated with weight-adjusted enoxaparin, antiplatelet agents, and drugs for ventricular remodelling.

The workup was further complemented by cardiac catheterization, which revealed coronary arteries without significant angiographic lesions (*Figure 1*).

Subsequently, cardiac magnetic resonance imaging was performed, confirming an EF of 46% with severe basal and medial anteroseptal hypokinesia and apical akinesia. An apical thrombus measuring 13 × 16 mm was identified, along with a circumferential subendocardial **late gadolinium enhancement (LGE) pattern in the apical and anteroseptal segments, involving 27% of the LV mass. T1 mapping sequences were increased at the medial septal and medial anteroseptal levels, and T2 STIR was positive for myocardial oedema (*Figure 3*; Supplementary material online, Videos S3–S5).

**Figure 3:**
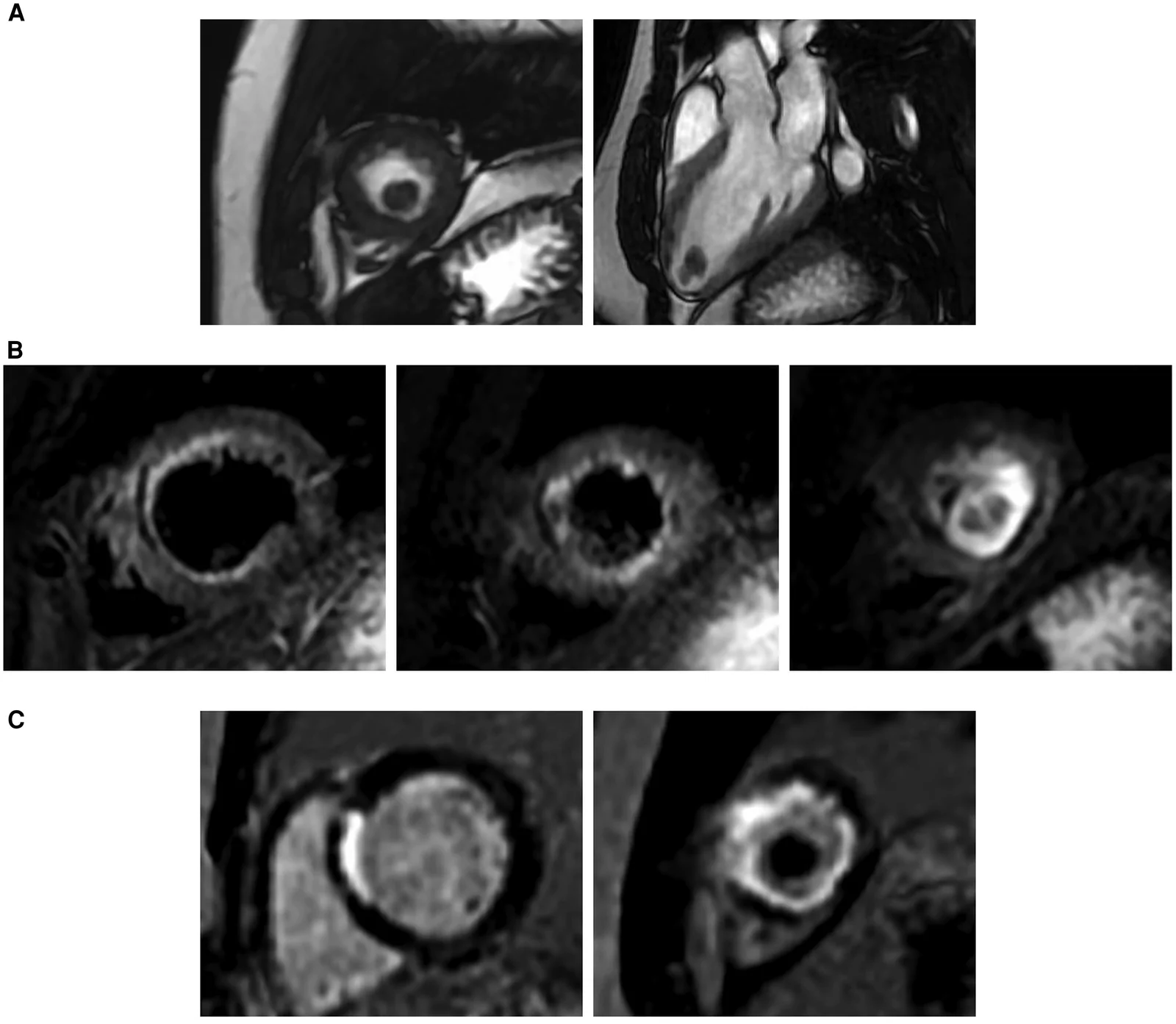
Cardiac magnetic resonance imaging. *(A)* Short-axis view at the apical level (left) and long-axis two-chamber view (right), showing a rounded image adjacent to the akinetic zone, suggestive of a thrombus. *(B)* T2 short tau inversion recovery (STIR) sequences, short-axis view at the medioapical level, showing hyperintense areas at the medial anteroseptal level and in the apical segments, suggestive of oedema (left and centre), and a rounded apical image with hyperintense areas suggestive of an acute thrombus (right). *(C)* Basal and medial anteroseptal subendocardial late enhancement and circumferential late enhancement in the apical segments with subendocardial involvement greater than 50% (left). A rounded apical image with negative late gadolinium enhancement is also observed, confirming the presence of an intracavitary thrombus (right).

After completing the extension studies and ruling out an ischaemic coronary event, the diagnosis of myocarditis was confirmed. Given the concomitant presence of peripheral eosinophilia, the clinical suspicion of eosinophilic myocarditis was established as the primary aetiology.

In the aetiological workup, extensive evaluation for secondary causes of eosinophilia, including parasitic, infectious, autoimmune, and haematologic disorders, was negative. Inflammatory markers such as C-reactive protein and erythrocyte sedimentation rate were negative. Serum immunoglobulin E (IgE) levels were markedly elevated (1562 mg/dL).

For a definitive diagnosis, a bone marrow biopsy was performed, which showed a slight increase in eosinophils, and an endomyocardial biopsy (EMB) was carried out with seven samples taken from the right ventricle, revealing mild fibrosis, an adherent fibrin-haematic thrombus, as well as myocyte necrosis with cellular debris. CD3 immunostaining was negative, leading to non-specific histopathological findings (*Figure 4*).

**Figure 4:**
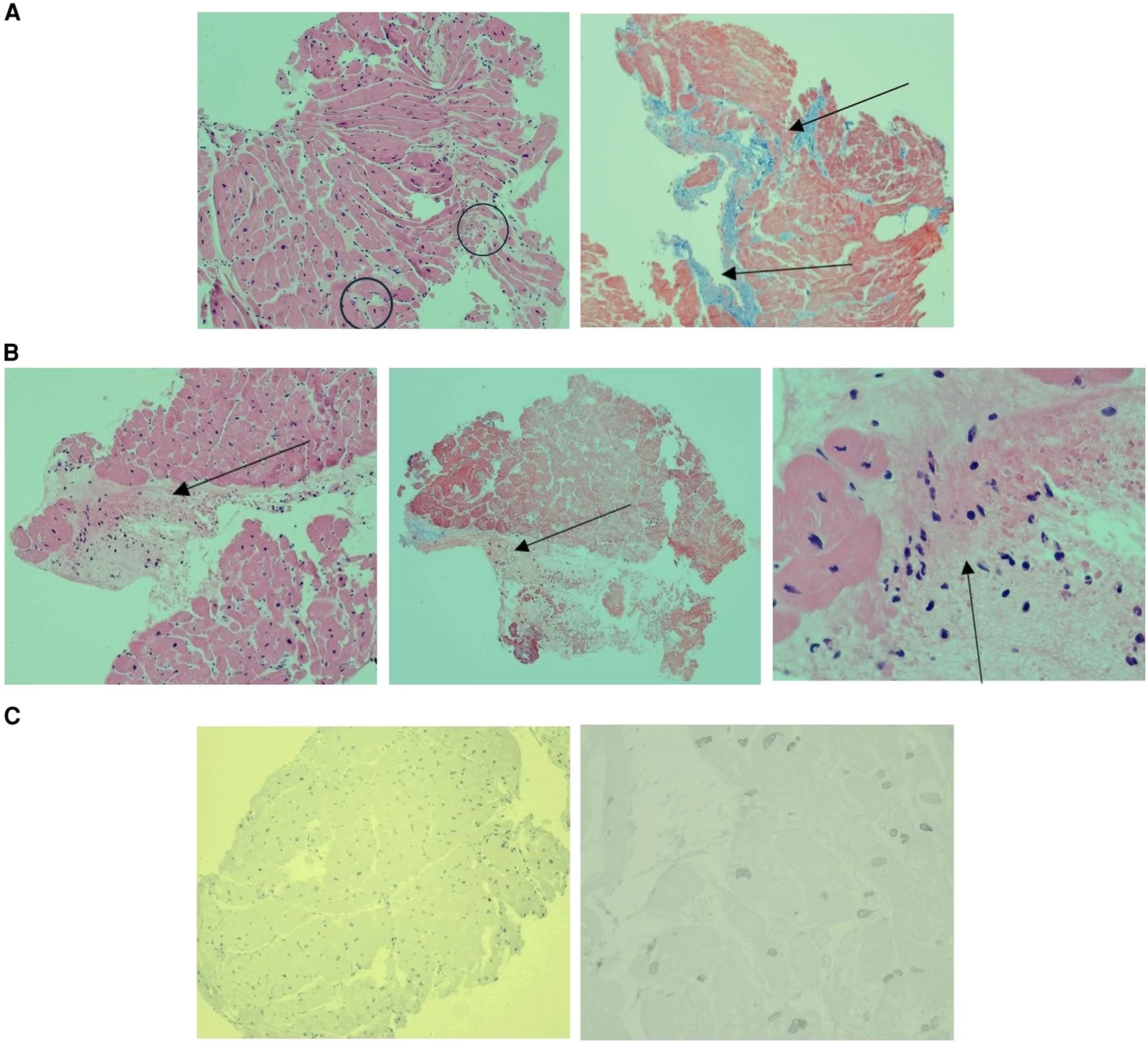
Endomyocardial biopsy of the right ventricle. *(A)* Haematoxylin and eosin staining (left): isolated myocyte necrosis and cellular debris. Masson’s trichrome staining (right): endocardial and interstitial fibrosis. *(B)* Haematoxylin and eosin staining: fibrin-haematic thrombus adherent to the endocardium. *(C)* Negative CD3 immunostaining.

Although the biopsy was non-specific, the samples were taken from the right-sided chambers, which resulted in lower sensitivity. The clinical suspicion of eosinophilic myocarditis remained strong; therefore, methylprednisolone pulses at 1 mg/kg/day were administered for 3 days, followed by high-dose oral meprednisone and prophylactic ivermectin, administered empirically to cover a possible parasitic infection in the setting of marked hypereosinophilia. The clinical course was favourable; an echocardiogram on 24 February demonstrated improvement of the LVEF to 50% and a marked reduction of the thrombus to 5 × 4 mm (*Figure 5*), allowing for outpatient follow-up under oral anticoagulation.

**Figure 5:**
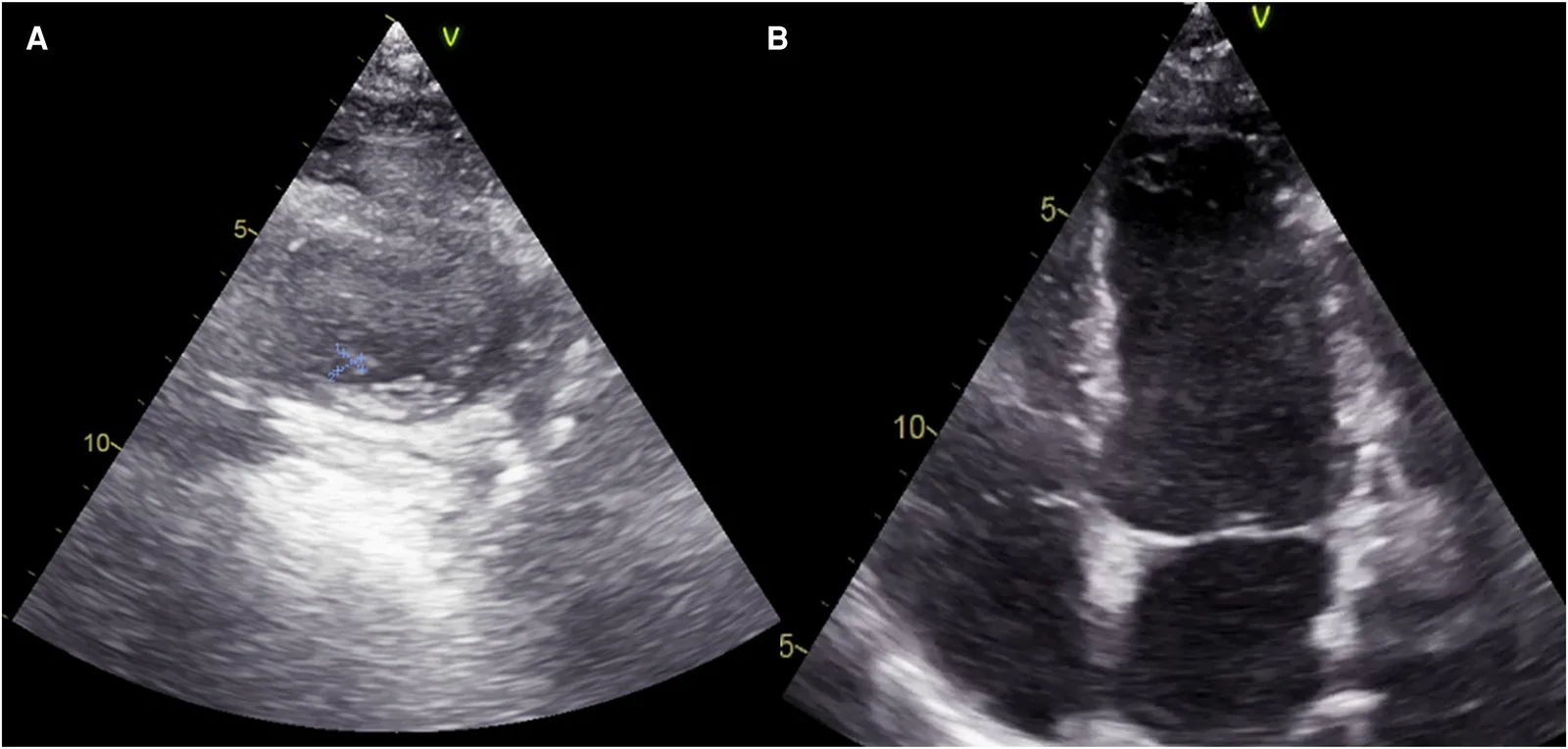
One-month follow-up echocardiogram. Parasternal short-axis echocardiographic view. A reduction in the intracavitary thrombus is observed, measuring 5 × 4 mm (left), not visualized in the apical four-chamber view (right).

## Discussion

Eosinophilic myocarditis is a rare but potentially life-threatening inflammatory cardiomyopathy in which delayed recognition may lead to severe complications, including heart failure and thromboembolic events.^1,2^

Clinically, it often mimics acute coronary syndrome, presenting with chest pain, electrocardiographic abnormalities, and elevated cardiac biomarkers.^1–3^ Therefore, it should be considered among the differential diagnoses of Myocardial Infarction With Non-obstructive Coronary Arteries, particularly in young patients without cardiovascular risk factors and with unexplained peripheral eosinophilia.^4^

Eosinophilic myocarditis may arise from diverse aetiologies, including primary haematologic disorders, secondary causes such as infections, drug hypersensitivity, or autoimmune diseases, and idiopathic forms.^1,3,5^ In the present case, extensive evaluation was negative, supporting an idiopathic origin.

Diagnosis relies on the integration of clinical, laboratory, and imaging findings. Among available modalities, cardiac magnetic resonance imaging has emerged as the most valuable non-invasive tool.^3,6^ The presence of myocardial oedema and circumferential subendocardial LGE represents a highly suggestive pattern, allowing differentiation from other forms of myocarditis.^3,6^

Although EMB remains the diagnostic gold standard, its sensitivity is limited by the focal and patchy distribution of inflammatory infiltrates, particularly when samples are obtained from the right ventricle while the predominant involvement affects the left ventricular subendocardium.^2,3^ Consequently, a negative biopsy does not exclude the diagnosis in the appropriate clinical setting.^2,3^

In this case, despite non-specific EMB findings, the diagnosis was strongly supported by the combination of marked peripheral eosinophilia and characteristic cardiac magnetic resonance features. Given the well-recognized limitations of EMB, including its reduced sensitivity due to the focal and patchy distribution of myocardial involvement, a negative result did not exclude eosinophilic myocarditis in this clinical context.^2,3^ Based on this integrated assessment, immunosuppressive therapy was initiated.

The rapid clinical and imaging improvement observed after corticosteroid therapy, including recovery of ventricular function and near-complete thrombus resolution, further reinforces the diagnosis. This case highlights that, in selected patients, a multimodal approach may be sufficient to establish the diagnosis and guide treatment, even in the absence of definitive histopathological confirmation.^1,2^

## Conclusion

Eosinophilic myocarditis is a complex condition that demands a high index of clinical suspicion, particularly in young patients presenting with chest pain and peripheral eosinophilia. As discussed, diagnostic delay driven by the non-specificity of symptoms may predispose to serious thrombotic complications and functional deterioration. However, the integration of tools such as cMRI and the early initiation of corticosteroid therapy allow for the reversal of myocardial damage and a dramatic improvement in patient prognosis. It is imperative that clinicians consider this entity in the differential diagnosis of non-atherosclerotic myocardial infarction to ensure timely intervention and prevent progression to end-stage heart failure.

## Lead author biography


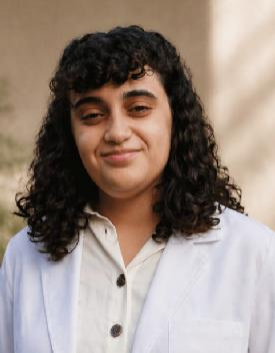


I am a physician and cardiology trainee at Hospital Universitario Austral, Buenos Aires, Argentina. My clinical and academic interests include heart failure, myocarditis, echocardiography, and multimodality cardiac imaging. I am actively involved in patient care, scientific writing, and cardiovascular research. I am committed to integrating evidence-based medicine into daily practice and to contributing to the diagnosis and management of complex cardiovascular diseases through research and education.

## Supplementary Material

ytag752_Supplementary_Data

## Data Availability

The data underlying this article are available in the article itself. Additional data are available from the corresponding author upon reasonable request.
